# Supplementary material for: MPCI: A novel metric for quantifying DNA methylation patterns in NGS data
Source: PLoS Comput Biol. 2026 Mar 24;22(3):e1014076. doi: 10.1371/journal.pcbi.1014076 (PMC13035127; doi:10.1371/journal.pcbi.1014076)
Supplement: S1 Table — (DOCX) [file pcbi.1014076.s005.docx]

**Supplementary Table 1: Comprehensive Performance Comparison of MPCI vs. Other Methylation Metrics on CD4 CD8 Classification Task**
*Values represent mean ± standard deviation from 10 repeated nested CV runs (5 outer folds, 3 inner folds)*

| Metric | AUC | AUC p-value vs MPCI | Accuracy | Accuracy p-value vs MPCI | Sensitivity | Sensitivity p-value vs MPCI | Specificity | Specificity p-value vs MPCI |
| --- | --- | --- | --- | --- | --- | --- | --- | --- |
| **MPCI** | **0.915 ± 0.15** | – | **0.740 ± 0.23** | – | **0.720 ± 0.32** | – | **0.760 ± 0.31** | – |
| qFDRP | 0.900 ± 0.17 | 0.646 (NS) | 0.655 ± 0.25 | 0.072 (NS) | 0.640 ± 0.37 | 0.274 (NS) | 0.670 ± 0.33 | 0.196 (NS) |
| uMHL | 0.885 ± 0.19 | 0.825 (NS) | 0.670 ± 0.23 | 0.472 (NS) | 0.700 ± 0.28 | 0.820 (NS) | 0.640 ± 0.42 | 0.107 (NS) |
| FDRP | 0.770 ± 0.21 | 0.0005 (***) | 0.530 ± 0.20 | 5.92×10⁻⁶ (***) | 0.480 ± 0.40 | 0.0018 (**) | 0.580 ± 0.34 | 0.0084 (***) |
| MHL | 0.700 ± 0.22 | 2.04×10⁻⁷ (***) | 0.470 ± 0.25 | 1.70×10⁻⁷ (***) | 0.470 ± 0.41 | 0.0013 (**) | 0.470 ± 0.32 | 1.46×10⁻⁵ (***) |
| EP | 0.700 ± 0.20 | 1.12×10⁻⁷ (***) | 0.455 ± 0.21 | 6.06×10⁻⁸ (***) | 0.410 ± 0.41 | 0.000135 (***) | 0.500 ± 0.34 | 0.000488 (***) |
| ME | 0.685 ± 0.22 | 2.57×10⁻⁸ (***) | 0.460 ± 0.26 | 3.74×10⁻⁸ (***) | 0.420 ± 0.39 | 0.000135 (***) | 0.500 ± 0.34 | 0.000331 (***) |
| PDR | 0.680 ± 0.19 | 1.91×10⁻⁸ (***) | 0.460 ± 0.20 | 3.74×10⁻⁸ (***) | 0.440 ± 0.40 | 0.000374 (***) | 0.480 ± 0.31 | 1.92×10⁻⁵ (***) |
| dMHL | 0.720 ± 0.21 | 8.61×10⁻⁶ (***) | 0.395 ± 0.24 | 8.27×10⁻¹⁰ (***) | 0.420 ± 0.41 | 4.08×10⁻⁵ (***) | 0.370 ± 0.31 | 3.97×10⁻⁷ (***) |
