# Supplementary material for: MPCI: A novel metric for quantifying DNA methylation patterns in NGS data
Source: PLoS Comput Biol. 2026 Mar 24;22(3):e1014076. doi: 10.1371/journal.pcbi.1014076 (PMC13035127; doi:10.1371/journal.pcbi.1014076)
Supplement: S2 Table — (DOCX) [file pcbi.1014076.s006.docx]

**Supplementary Table 2: Analysis on MHB regions on CD4 CD8 Classification Task**
 **(*chr22 only, for computational efficiency*):**

| **Metric** | **MPCI (Mean)** | **MHL (Mean)** | **Difference** | **p-value** |
| --- | --- | --- | --- | --- |
| **AUC** | 0.975 | 0.905 | +0.070 | 0.027 (*) |
| **Accuracy** | 0.905 | 0.860 | +0.045 | 0.158 (NS) |
| **Sensitivity** | 1.000 | 0.930 | +0.070 | 0.006 (**) |
| **Specificity** | 0.810 | 0.790 | +0.020 | 0.688 (NS) |
