# Supplementary material for: MPCI: A novel metric for quantifying DNA methylation patterns in NGS data
Source: PLoS Comput Biol. 2026 Mar 24;22(3):e1014076. doi: 10.1371/journal.pcbi.1014076 (PMC13035127; doi:10.1371/journal.pcbi.1014076)
Supplement: S3 Table — (DOCX) [file pcbi.1014076.s007.docx]

**Supplementary Table 3: Performance Comparison of MPCI, dMHL, and MHL Across Spike-in Ratios (Mean ± SD)**

| **Spike_Ratio** | **Metric** | **AUC** | **Sensitivity** | **Specificity** | **Accuracy** |
| --- | --- | --- | --- | --- | --- |
| **1** | **dMHL** | 0.56 ± 0.06 | 0.49 ± 0.14 | 0.47 ± 0.13 | 0.48 ± 0.07 |
|  | **MPCI** | 0.57 ± 0.06 | 0.48 ± 0.13 | 0.49 ± 0.14 | 0.49 ± 0.08 |
|  | **MHL** | 0.56 ± 0.06 | 0.5 ± 0.14 | 0.46 ± 0.13 | 0.48 ± 0.07 |
| **2** | **dMHL** | 0.67 ± 0.09 | 0.62 ± 0.12 | 0.62 ± 0.11 | 0.62 ± 0.08 |
|  | **MPCI** | 0.69 ± 0.09 | 0.63 ± 0.12 | 0.64 ± 0.1 | 0.64 ± 0.08 |
|  | **MHL** | 0.62 ± 0.09 | 0.57 ± 0.13 | 0.58 ± 0.13 | 0.58 ± 0.09 |
| **3** | **dMHL** | 0.8 ± 0.08 | 0.71 ± 0.12 | 0.73 ± 0.11 | 0.72 ± 0.08 |
|  | **MPCI** | 0.82 ± 0.06 | 0.74 ± 0.11 | 0.74 ± 0.11 | 0.74 ± 0.06 |
|  | **MHL** | 0.74 ± 0.09 | 0.66 ± 0.12 | 0.69 ± 0.11 | 0.67 ± 0.08 |
| **4** | **dMHL** | 0.91 ± 0.06 | 0.82 ± 0.11 | 0.84 ± 0.1 | 0.83 ± 0.08 |
|  | **MPCI** | 0.92 ± 0.04 | 0.83 ± 0.08 | 0.85 ± 0.08 | 0.84 ± 0.05 |
|  | **MHL** | 0.86 ± 0.06 | 0.77 ± 0.1 | 0.78 ± 0.1 | 0.78 ± 0.07 |
| **5** | **dMHL** | 0.92 ± 0.04 | 0.82 ± 0.08 | 0.87 ± 0.08 | 0.84 ± 0.06 |
|  | **MPCI** | 0.96 ± 0.03 | 0.9 ± 0.07 | 0.91 ± 0.06 | 0.91 ± 0.04 |
|  | **MHL** | 0.9 ± 0.05 | 0.81 ± 0.09 | 0.82 ± 0.09 | 0.82 ± 0.06 |
| **10** | **dMHL** | 1 ± 0.01 | 0.95 ± 0.05 | 0.98 ± 0.04 | 0.96 ± 0.03 |
|  | **MPCI** | 1 ± 0 | 0.98 ± 0.03 | 0.99 ± 0.02 | 0.99 ± 0.02 |
|  | **MHL** | 0.99 ± 0.01 | 0.94 ± 0.06 | 0.97 ± 0.04 | 0.95 ± 0.04 |
