# Supplementary material for: MPCI: A novel metric for quantifying DNA methylation patterns in NGS data
Source: PLoS Comput Biol. 2026 Mar 24;22(3):e1014076. doi: 10.1371/journal.pcbi.1014076 (PMC13035127; doi:10.1371/journal.pcbi.1014076)
Supplement: Attachment — Submitted filename: 01-28-2026_Response to Reviewers.docx [file pcbi.1014076.s009.docx]

Response to editor:

Thank you for the opportunity to revise our manuscript for *PLOS Computational Biology*. We have carefully addressed all the technical points and formatting requirements outlined in your letter. Below is our point-by-point response. All changes have been implemented in the revised manuscript and supporting files.

1) Please ensure that the CRediT author contributions listed for every co-author are completed accurately and in full. At this stage, the following Authors/Authors require contributions: Hoda Mohammadzade, Naghme Nazer, and Mahya Mehrmohamadi. Please ensure that the full contributions of each author are acknowledged in the "Add/Edit/Remove Authors" section of our submission form. The list of CRediT author contributions may be found here: [https://journals.plos.org/ploscompbiol/s/authorship#loc-author-contributions](https://journals.plos.org/ploscompbiol/s/authorship%23loc-author-contributions)

We have updated the "Author Contributions" section in the manuscript to fully detail the roles of each author using the CRediT taxonomy. These contributions have also been accurately reflected in the online submission form.

2) Please provide an Author Summary. This should appear in your manuscript between the Abstract (if applicable) and the Introduction, and should be 150-200 words long. The aim should be to make your findings accessible to a wide audience that includes both scientists and non-scientists. Sample summaries can be found on our website under Submission Guidelines: [https://journals.plos.org/ploscompbiol/s/submission-guidelines#loc-parts-of-a-submission](https://journals.plos.org/ploscompbiol/s/submission-guidelines%23loc-parts-of-a-submission)

Author Summary has been added to the main manuscript between the abstract and introduction.

3) Some material included in your submission may be copyrighted. According to PLOSu2019s copyright policy, authors who use figures or other material (e.g., graphics, clipart, maps) from another author or copyright holder must demonstrate or obtain permission to publish this material under the Creative Commons Attribution 4.0 International (CC BY 4.0) License used by PLOS journals. Please closely review the details of PLOSu2019s copyright requirements here: PLOS Licenses and Copyright. If you need to request permissions from a copyright holder, you may use PLOS's Copyright Content Permission form. Please respond directly to this email and provide any known details concerning your material's license terms and permissions required for reuse, even if you have not yet obtained copyright permissions or are unsure of your material's copyright compatibility. Once you have responded and addressed all other outstanding technical requirements, you may resubmit your manuscript within Editorial Manager. Potential Copyright Issues: i) Figures 1A, 2A, 3A, and 4A. Please confirm whether you drew the images / clip-art within the figure panels by hand. If you did not draw the images, please provide (a) a link to the source of the images or icons and their license / terms of use; or (b) written permission from the copyright holder to publish the images or icons under our CC BY 4.0 license. Alternatively, you may replace the images with open source alternatives. See these open source resources you may use to replace images / clip-art: - https://commons.wikimedia.org - <https://openclipart.org/>.

We have now regenerated all figures (including 1A, 2A, 3A, and 4A) by hand and have completely removed any previously used non-original icons or clip-art. All graphical elements in these figures are now original content created by the authors. These revised figures are original to this work and are fully compliant with PLOS’s CC BY 4.0 license requirements.

4) In the online submission form, you indicated that cross-validation, and spike-in process can be shared upon request.. All PLOS journals now require all data underlying the findings described in their manuscript to be freely available to other researchers, either 1. In a public repository 2. Within the manuscript itself 3. Uploaded as supplementary information. This policy applies to all data except where public deposition would breach compliance with the protocol approved by your research ethics board. If your data cannot be made publicly available for ethical or legal reasons (e.g., public availability would compromise patient privacy), please explain your reasons by return email and your exemption request will be escalated to the editor for approval. Your exemption request will be handled independently and will not hold up the peer review process, but will need to be resolved should your manuscript be accepted for publication. One of the Editorial team will then be in touch if there are any issues.

All datasets used in this study are publicly available from the GEO database under the accession codes referenced in the manuscript (GSE186458, GSE262275, GSE43414). All custom R scripts used for data analysis, simulation, metric calculation (including the MPCI function), and figure generation are publicly available under an open-source license at:
<https://github.com/NaghmeNazer/MPCI>

5) Please amend your detailed Financial Disclosure statement. This is published with the article. It must therefore be completed in full sentences and contain the exact wording you wish to be published. 1) State what role the funders took in the study. If the funders had no role in your study, please state: "The funders had no role in study design, data collection and analysis, decision to publish, or preparation of the manuscript." 2) If any authors received a salary from any of your funders, please state which authors and which funders. If you did not receive any funding for this study, please simply state: u201cThe authors received no specific funding for this work.u201d

The financial disclosure has been amended to the required full-sentence format and now reads:
*“We thank the University of Tehran (UT) and Sharif University of Technology (SUT) for server and computing resources. The study is funded by Iran National Science Foundation, grant number 99012262 and Research and Technology Office of Sharif University of Technology (SUT). The funders had no role in study design, data collection and analysis, decision to publish, or preparation of the manuscript.”*

**Reviewers' comments:**

Reviewer's Responses to Questions

**Comments to the Authors:
Please note here if the review is uploaded as an attachment.**

Reviewer #1: DNA methylation abnormality is vital to many diseases. Due to the nature of DNA methylation, which would only occur in CpG sites, focusing on methylation haplotype rather than individual sites can be more beneficial in consistently distinguishing between the healthy and abnormal methylation profiles. The author introduced a new metric MPCI which overcome the disadvantages of current metrics such as MHL and showed more superior performance in distinguishing different cell types as well as better disease detection ability from simulated cfDNA.

We appreciate the reviewer’s careful consideration of our work. We have addressed all their insightful comments in the revised version of the manuscript to the best of our ability and believe the revised manuscript has thus improved in quality and robustness.

**Major comments:**

What is the rationale of summing up the MS(R) and MS(RT)? I understand that the author want to also consider the consistency among different reads. But since the distance for rows represent the similarity among different reads and the distance for columns represent the similarity among the CpG sites, it seems not distinguishing between the difference of the different CpG sites in the candidate region like what MHL does. Is there any biological evidence that can prove that small portions of reads with consecutive CpGs should be considered the same level of methylation the same as the randomly methylated ones? (as the Figure 1 showed)

We thank the reviewer for this insightful question regarding the biological rationale for combining row and column similarities in MPCI. The core of our method is designed to be highly sensitive to the presence of small, consistent haplotypes, which is crucial for rare haplotype detection in diagnostic contexts e.g. liquid biopsy applications. We agree that biologically, stable, inherited methylation haplotypes are typically characterized by consecutive blocks of methylation. MPCI is indeed tuned to prioritize these strong, consecutive signals.

Here, we explain in detail how MPCI achieves this (this explanation in now added to the discussion section of the revised manuscript for further clarification):

1. The Manhattan similarity is maximized for identical, consecutive patterns. A read pair like `[1,1,1,1,1,1]` and `[1,1,1,1,1,1]` achieves perfect similarity, directly rewarding consistent haplotypes and always gets a weight of 1 during the final averaging.

2. The weighting scheme (W_i,j) de-prioritizes random noise. Reads with a balanced number of 1s and 0s (e.g., `[1,0,1,0,1,0]`), which are indicative of random, non-consecutive methylation, receive a random 1 or -1 weight. This ensures that coincidentally matching but biologically implausible "patterns" contribute randomly and thus less to the final MPCI score, preventing them from being valued equally to true consecutive haplotypes. However , repeating signals from inherited haplotypes that may not be **fully** methylated from end-to-end but still exhibit a consistent, non-random pattern across multiple reads can show a meaningful biological incidence which is worth considering in the final MPCI score.

At the same time, a key innovation of MPCI is its sensitivity to the exact start and end points of a pattern, which MHL overlooks as it scans for consecutive methylated substrings anywhere within a read. MPCI captures biologically significant, haplotype-derived repeated signals even when they are not fully methylated end-to-end. This allows it to detect a broader and more realistic range of consistent methylation patterns that are informative for tissue tracing thus enhances its ability to detect low-abundance, tissue-derived cfDNA haplotype, where any consistent signature—even if not perfectly consecutive across all CpGs—can be highly informative amidst the vast background of different haplotypes.

The Figure 4 is a little cherry-picked to me. Is there any example where the MHL can better distinguish than MPCI for some DMRs? Also since the definition of MHL and MPCI is different and the range is also different, it seems to be not intuitive and convincing to directly compare the absolute difference of the exact values.

We agree with the reviewer that direct comparison of raw values derived from metrics with different scales is not valid. To address this concern, we have now normalized the differential scores of both MPCI and MHL to 0–2 scale for all prioritization analyses (Figures 4 and 5). This normalization enables a fair and intuitive comparison of their relative ranking performance, which is the primary objective of this analysis. The manuscript text and figures have been updated accordingly.

To select representative regions for plotting, we generated a scatter plot comparing the MPCI and MHL differential values for all input DMRs. As anticipated, the majority of DMRs showed agreement between MHL and MPCI, forming a clear linear relationship (Figure 1). We subsequently divided the scatter plot into four distinct quadrants:

- **Region 1**: Markers with high MPCI but low MHL differential scores. These 51 markers are prioritized by MPCI but overlooked by MHL.
- **Region 2**: Markers with both high MPCI and high MHL scores. These 36 markers are top-ranked by both metrics.
- **Region 3**: Markers with low differential scores for both MPCI and MHL. These 939 markers are consistently assigned low priority by both methods.
- **Region 4**: Markers with low MPCI but high MHL scores. These 6 markers are prioritized by MHL but not by MPCI.

We next focused on DMRs in Region1 and Region4 that fall off of the diagonal line. For each of such outliers, we examined neighboring regions along the vertical and horizontal directions of the scatter plot. Specifically, when a region exhibited a large deviation in MHL, we analyzed other regions with similar MPCI values but differing MHL scores. This approach allows us to investigate why a particular region receives a high priority (large difference) by one metric but not the other, and to understand the underlying differences in how MPCI and MHL score regions.

**Figure1: Comparison Scheme**

In Figure 1, the most outlier marker in region1 is selected (Marker1). Thus, the orange points highlight two markers with similar MPCI differential values (1.45) but substantially different MHL differential values (Marker1 = 0.41, Marker2 = 1.56). Visualization of these regions (Figure 2 and Figure 3) shows that both markers exhibit similar methylation patterns and same prioritization score can be rational for them. It should be noted that the difference in MHL is partially driven by noisy unmethylated signals in Marker1, which disrupt fully methylated haplotypes and are therefore penalized by the MHL formulation. Importantly, Marker1 is a reasonable candidate as figure 3 shows. However, its small MHL score difference (0.41) classifies it as a weak marker and can exclude it entirely which can be a false exclusion of a good candidate region. MPCI assigns identical ranks to both regions.

In Figure 1, the most prominent outlier in Region 1 was selected for further analysis (Marker1). The orange circles illustrates two markers with nearly identical MPCI differential values (~1.45) but vastly different MHL differential values (Marker1 = 0.41, Marker2 = 1.56).

Visual inspection of their methylation patterns (Figure 2 and Figure 3) reveals that both markers are highly similar, justifying the identical prioritization score assigned by MPCI. The discrepancy in MHL scores arises because Marker1 contains noisy unmethylated signals within its region. These signals disrupt the detection of fully methylated haplotypes, a requirement heavily weighted by the MHL metric, thereby penalizing Marker1.

Critically, as shown in Figure 3, Marker1 appears as a plausible candidate. However, its low MHL score of 0.41 categorizes it as a weak marker, which risks its complete exclusion from consideration—a potential false negative. In contrast, MPCI assigns both regions an identical, high-priority, capturing the true similarity that MHL overlooks.

**Figure2: Methylation Reads in Marker1 (MHL Diff = 0.41, MPCI Diff = 1.45)**

**Figure3: Methylation Reads in Marker2 (MHL Diff = 1.56, MPCI Diff = 1.45)**

Conversely, MarkerX represents the most significant outlier in Region 4. Upon examining the sequencing reads, we found that cfDNA samples contained either zero or a maximum of two reads covering this region. This extreme lack of coverage suggests that MarkerX (chr1:143916766- 143917144 (hg19)) is a likely false positive selection by MHL, as its score is derived from statistically insufficient data (Average coverage in 23 cfDNA samples = 0.48X).

We therefore investigated the second-most prominent outlier in this region, Marker3. Here, the red circles highlight two markers (Marker3 and Marker4) that receive nearly identical MHL scores but are ranked very differently by MPCI. Visual inspection confirms that Marker4 is the superior candidate, consistent with its higher MPCI ranking (Figure 4 and Figure 5).

The discrepancy arises from the specific behavior of Marker3: it shows a mosaic of partial methylation and unmethylation in the neuronal sample, but appears fully methylated in the cfDNA sample. This pattern results in a very high MHL value in cfDNA (near 1) and a low value in neurons, producing a large—but misleading—MHL differential score. This represents another instance of a false positive prioritization by MHL.

In this case, MPCI's assignment of a score below 1 to Marker3 rationally reflects its compromised quality as a marker, demonstrating its robustness against such artifacts.

**Figure4: Methylation Reads in Marker3 (MHL Diff = 1.27, MPCI Diff = 0.81)**

**Figure5: Methylation Reads in Marker4 (MHL Diff = 1.25, MPCI Diff = 1.63)**

Overall, we believe that MPCI ranking for marker prioritization is more robust. We have updated the results section of the revised manuscript to include this flow of analysis and updated Figure 4. We thank the reviewer for this question as we think with this update, the validity of our conclusion has improved.

MHL is sensitive to the length of the considered region and could be underestimated if there are more NAs and length is large. When selecting the CpG with different numbers, do they overlap with each other? For a specific region, how to determine what CpG number is the best?

**We thank the reviewer for these important questions regarding our region selection.**

1. **Regarding region overlap:** We confirm that the 400 genomic regions were selected to be non-overlapping to ensure the independence of data points for our classifier. In our post-selection validation, we found that 392 regions were entirely independent. A minimal overlap was detected in 8 regions (constituting 4 pairs), affecting only 32 out of 4,958 total CpG sites (0.65%). We have verified that removing these 8 overlapping regions does not change our performance conclusions, confirming that our results are robust and not biased by regional dependencies. However all analyses were repeated and updated using the non overlapping regions and this is mentioned in our revised manuscript (Methods and Results section).
2. **Regarding the "best" CpG number:** We deliberately selected regions with a wide range of CpG counts (3 to 22) to demonstrate that **MPCI's performance is robust and not dependent on a specific, pre-defined region size (Methods section “Data collection and preprocessing).** A key finding of our study is that MPCI works effectively across various genomic contexts and region lengths. Therefore, there is no single "best" CpG number; users can apply MPCI to regions of different sizes relevant to their biological question without needing to pre-optimize for length. This flexibility is a practical advantage of our metric.

The author described some other metrics than the MHL, have you compared these metrics with MPCI?

We thank the reviewer for raising this important point. In response, we have conducted an extensive and statistically rigorous comparison of MPCI against 7 DNA methylation quantification metrics:

- Methylation Haplotype Load (MHL)
- Unmethylation Haplotype Load (uMHL)
- Methylation Entropy (ME)
- Epi-polymorphism (EP)
- Proportion of Discordant Reads (PDR)
- Fraction of Discordant Read Pairs (FDRP)
- Qunatitative FDRP (qFDRP)

The evaluation was performed on the same CD4 vs. CD8 classification task under identical conditions (linear SVM kernel, identical training/test splits, and feature preprocessing). To ensure robustness and prevent overfitting, we implemented a nested cross-validation strategy with:

- 5 outer folds and 3 inner folds for hyperparameter tuning (cost parameter C for linear SVM),

- 10 independent repetitions to account for variability,

- Statistical significance assessed via nonparametric Wilcoxon rank-sum tests between all metric pairs.

Our comprehensive results demonstrate that:

1. MPCI achieves the highest mean AUC (0.915 ± 0.15) among all other metrics. It reaches statistically significant improvement in comparison with all metrics except qFDRP and uMHL (Table 1).

2. Across all performance metrics (**Accuracy, Sensitivity, Specificity**), MPCI consistently ranks the top performer (Table 2). Specifically, MPCI achieves the highest Accuracy (0.740), Sensitivity (0.720), and Specificity (0.760).

**Table 1: AUC Performance Comparison (Sorted by Mean AUC)**

| Metric | Mean_AUC | SD_AUC | vs. MPCI (p-value) | Significance |
| --- | --- | --- | --- | --- |
| MPCI | 0.915 | 0.15 | – | – |
| qFDRP | 0.900 | 0.17 | 0.646 | NS |
| uMHL | 0.885 | 0.19 | 0.825 | NS |
| FDRP | 0.770 | 0.21 | 0.0005 | *** |
| MHL | 0.700 | 0.22 | 2.04×10⁻⁷ | *** |
| EP | 0.700 | 0.20 | 1.12×10⁻⁷ | *** |
| ME | 0.685 | 0.22 | 2.57×10⁻⁸ | *** |
| PDR | 0.680 | 0.19 | 1.91×10⁻⁸ | *** |

**Table 2: Comprehensive Performance Comparison of MPCI vs. Other Methylation Metrics**
*Values represent mean ± standard deviation from 10 repeated nested CV runs (5 outer folds, 3 inner folds)*

| Metric | AUC | AUC p-value vs MPCI | Accuracy | Accuracy p-value vs MPCI | Sensitivity | Sensitivity p-value vs MPCI | Specificity | Specificity p-value vs MPCI |
| --- | --- | --- | --- | --- | --- | --- | --- | --- |
| **MPCI** | **0.915 ± 0.15** | – | **0.740 ± 0.23** | – | **0.720 ± 0.32** | – | **0.760 ± 0.31** | – |
| qFDRP | 0.900 ± 0.17 | 0.646 (NS) | 0.655 ± 0.25 | 0.072 (NS) | 0.640 ± 0.37 | 0.274 (NS) | 0.670 ± 0.33 | 0.196 (NS) |
| uMHL | 0.885 ± 0.19 | 0.825 (NS) | 0.670 ± 0.23 | 0.472 (NS) | 0.700 ± 0.28 | 0.820 (NS) | 0.640 ± 0.42 | 0.107 (NS) |
| FDRP | 0.770 ± 0.21 | 0.0005 (***) | 0.530 ± 0.20 | 5.92×10⁻⁶ (***) | 0.480 ± 0.40 | 0.0018 (**) | 0.580 ± 0.34 | 0.0084 (***) |
| MHL | 0.700 ± 0.22 | 2.04×10⁻⁷ (***) | 0.470 ± 0.25 | 1.70×10⁻⁷ (***) | 0.470 ± 0.41 | 0.0013 (**) | 0.470 ± 0.32 | 1.46×10⁻⁵ (***) |
| EP | 0.700 ± 0.20 | 1.12×10⁻⁷ (***) | 0.455 ± 0.21 | 6.06×10⁻⁸ (***) | 0.410 ± 0.41 | 0.000135 (***) | 0.500 ± 0.34 | 0.000488 (***) |
| ME | 0.685 ± 0.22 | 2.57×10⁻⁸ (***) | 0.460 ± 0.26 | 3.74×10⁻⁸ (***) | 0.420 ± 0.39 | 0.000135 (***) | 0.500 ± 0.34 | 0.000331 (***) |
| PDR | 0.680 ± 0.19 | 1.91×10⁻⁸ (***) | 0.460 ± 0.20 | 3.74×10⁻⁸ (***) | 0.440 ± 0.40 | 0.000374 (***) | 0.480 ± 0.31 | 1.92×10⁻⁵ (***) |

These results have been integrated into the revised manuscript in the Results section (Performance of MPCI in biomarker discovery contexts). Bar plot of auc comparison is added to figure 2 panel b. Details of other classification metrics is uploaded as Supplementary Table 1. PCA plots of all metrics is attached as Supplementary Figure 1.

To further evaluate the MPCI vs MHL/uMHL, we have defined dMHL = MHL-uMHL as reviewer #2 suggested. We observed that dMHL is aligned in direction with MPCI and, like MPCI, simultaneously reflects methylated and unmethylated states, making it a more appropriate comparator than MHL or uMHL alone. We have therefore reanalyzed all comparison experiments using dMHL in the revision. Regarding the classification task of CD4 vs. CD8, MPCI shows superior performance (Table 3). As can be seen in Table 2 and Table 3, dMHL performs better than MHL in classification of CD4 and CD8 samples as we expected. These results have been added into the revised manuscript in the Results section (Performance of MPCI in biomarker discovery contexts). Bar plot of classification metrics’ comparison is added to Fig 2c.

**Table 3: Comprehensive Performance Comparison of MPCI vs. dMHL (= MHL – uMHL)**
*Values represent mean ± standard deviation from 10 repeated nested CV runs (5 outer folds, 3 inner folds)*

| Metric | AUC | AUC p-value vs MPCI | Accuracy | Accuracy p-value vs MPCI | Sensitivity | Sensitivity p-value vs MPCI | Specificity | Specificity p-value vs MPCI |
| --- | --- | --- | --- | --- | --- | --- | --- | --- |
| MPCI | 0.915 ± 0.15 | – | 0.740 ± 0.23 | – | 0.720 ± 0.32 | – | 0.760 ± 0.31 | – |
| dMHL | 0.720 ± 0.21 | 8.61×10⁻⁶ (***) | 0.395 ± 0.24 | 8.27×10⁻¹⁰ (***) | 0.420 ± 0.41 | 4.08×10⁻⁵ (***) | 0.370 ± 0.31 | 3.97×10⁻⁷ (***) |

Reviewer #2: Nazer et al. developed a simple yet effective metric, MPCI, to quantify DNA methylation patterns in regions where nearby CpGs are concordantly methylated or unmethylated. The authors demonstrated that MPCI outperforms existing metrics, particularly MHL, which only quantifies the level of co-methylation. The manuscript presents interesting findings, but the following issues should be addressed before it can be considered for publication in PLoS Computational Biology:

We appreciate the reviewer’s time and insights. We believe the revised manuscript has improved significantly after addressing the reviewer’s comments.

(1) The schematic plots in Figures 1B and 1C appear to be inconsistent. If M represents mean methylation in Figure 1B, it should equal 1 for the control region. Similarly, beta in Figure 1C should equal 0 for the control region. Please verify and correct these illustrations.

We thank the reviewer for their precise attention to detail. The reviewer is correct. We have verified this error and have now corrected the values in both Figures 1B and 1C in the revised manuscript. We apologize for this oversight.

(2) MPCI appears to be mathematically equivalent to (MHL - uMHL). If we define dMHL = MHL - uMHL, how does its performance compare to MPCI? I recommend including dMHL in all comparative figures and providing a scatter plot showing the relationship between MPCI and dMHL.

We thank the reviewer for this insightful comment on the relationship between MPCI and the difference between MHL and uMHL (dMHL). Indeed, since $\text{dMHL}=\text{MHL}-\text{uMHL}$, it can be viewed as a simple symmetric extension of the original MHL metric. However, MPCI is not mathematically equivalent to dMHL. Rather, it is a distinct measure derived from weighted Manhattan similarities across reads and CpG sites, specifically designed as a single metric to capture methylation pattern consistency in a symmetric manner for both methylated and unmethylated states.

We confirm that dMHL is aligned in direction with MPCI and, like MPCI, simultaneously reflects methylated and unmethylated states, making it a more appropriate comparator than MHL or uMHL alone. Following the reviewer’s suggestion, we have now reanalyzed all comparison experiments using dMHL. We believe this change substantially strengthens the benchmarking analysis, and we sincerely thank the reviewer for this valuable and constructive suggestion.

Specifically to address the reviewer's question, we compared the performance of MPCI and dMHL in the same CD4 vs. CD8 classification task. To ensure robust model evaluation and mitigate overfitting risks, we employed a **nested cross-validation framework** comprising:

- **5 outer folds** and **3 inner folds** for hyperparameter optimization (cost parameter *C* for linear SVM),
- **10 independent repetitions** to account for stochastic variability,
- **Nonparametric Wilcoxon ranksum tests** to assess statistical significance across all metric pairs.

The results demonstrate that MPCI significantly outperforms dMHL across all performance metrics:

**Table 4: Comprehensive Performance Comparison of MPCI vs. dMHL (= MHL – uMHL)**
*Values represent mean ± standard deviation from 10 repeated nested CV runs (5 outer folds, 3 inner folds)*

| **Metric** | **MPCI (Mean ± SD)** | **dMHL (Mean ± SD)** | **Difference** | **p-value** |
| --- | --- | --- | --- | --- |
| **AUC** | 0.915 ± 0.15 | 0.720 ± 0.21 | +0.195 | 8.61×10⁻⁶ (***) |
| **Accuracy** | 0.740 ± 0.23 | 0.395 ± 0.24 | +0.345 | 8.27×10⁻¹⁰ (***) |
| **Sensitivity** | 0.720 ± 0.32 | 0.420 ± 0.41 | +0.300 | 4.08×10⁻⁵ (***) |
| **Specificity** | 0.760 ± 0.31 | 0.370 ± 0.31 | +0.390 | 3.97×10⁻⁷ (***) |

The performance gap is particularly pronounced in Accuracy and Specificity, where MPCI shows improvements of 0.345 and 0.390, respectively. These differences are highly statistically significant (all p < 0.001).

Key distinctions between MPCI and dMHL:

1. Mathematical formulation: MPCI is based on weighted Manhattan similarities computed across reads and CpG sites, whereas dMHL is simply the arithmetic difference between MHL and uMHL.

2. Handling of partial methylation patterns: MPCI accounts for consistency within each read and across reads simultaneously, while dMHL aggregates two separate metrics that each focus only on fully methylated or fully unmethylated haplotypes.

3. Symmetric weighting: MPCI assigns weights based on the proportion of methylated/unmethylated CpGs in pairwise comparisons, providing a more balanced representation of methylation consistency.

These results confirm that MPCI is not only distinct from dMHL in formulation but also substantially superior in discriminative power for detecting methylation differences between closely related cell types. The comparative analysis has been included in the revised manuscript as part of Results section (Performance of MPCI in biomarker discovery contexts – definition of differential MHL (dMHL) – Figure 2c). We have also added dMHL values to figure 1 for our schematic regions.

Following the reviewer's suggestion, we have further examined the relationship between MPCI and dMHL values by generating scatter plots for CD4 and CD8 cells separately (Figure 5). This scatter plot is added to the paper as Supplementary Figure 3. The analysis reveals a positive correlation between the two metrics (R² = 0.81, p < 0.001), confirming that while they share some statistical association, they are not mathematically equivalent. The observed correlation supports the intuitive notion that both metrics capture aspects of methylation symmetry. However, the unexplained variance and systematic deviations highlight that MPCI incorporates information not captured by the dMHL and vice versa. These differences translate to the substantial performance gap demonstrated in our classification experiments, where MPCI consistently outperforms dMHL across all evaluation metrics.

**Figure 5: Scatter plot of MPCI vs dMHL values for CD4 and CD8 cell types**

In the context of simulated spikes we conducted a detailed reanalysis comparing all three metrics (MPCI, dMHL, and MHL) using our spike-in simulation framework.

**Method:** The analysis was performed using our previously described spike-in simulation with five independent replicates per condition. Performance was evaluated using nested-cv SVM classification with a linear kernel. All results are reported as the mean ± standard deviation.

**Results**: As the reviewer correctly noted, the performance difference between MPCI and dMHL is indeed less pronounced than that between MPCI and MHL. This is evident in the table 5, where the numerical gap between MPCI and dMHL values is consistently smaller than the gap between MPCI and MHL values across all spike-in ratios. We have incorporated a discussion of these comparative results in the revised manuscript (Results section - Performance of MPCI in simulated disease detection contexts). Also the line plot of comparison of auc values is added to Fig 3b. The full results of classification is also uploaded as Supplementary Table 3. Thanks for raising this point, which allowed us to provide a more complete analysis.

**Table 5: Performance Comparison of MPCI, dMHL, and MHL Across Spike-in Ratios (Mean ± SD)**

| **Spike_Ratio** | **Metric** | **AUC** | **Sensitivity** | **Specificity** | **Accuracy** |
| --- | --- | --- | --- | --- | --- |
| **1** | **dMHL** | 0.56 ± 0.06 | 0.49 ± 0.14 | 0.47 ± 0.13 | 0.48 ± 0.07 |
|  | **MPCI** | 0.57 ± 0.06 | 0.48 ± 0.13 | 0.49 ± 0.14 | 0.49 ± 0.08 |
|  | **MHL** | 0.56 ± 0.06 | 0.5 ± 0.14 | 0.46 ± 0.13 | 0.48 ± 0.07 |
| **2** | **dMHL** | 0.67 ± 0.09 | 0.62 ± 0.12 | 0.62 ± 0.11 | 0.62 ± 0.08 |
|  | **MPCI** | 0.69 ± 0.09 | 0.63 ± 0.12 | 0.64 ± 0.1 | 0.64 ± 0.08 |
|  | **MHL** | 0.62 ± 0.09 | 0.57 ± 0.13 | 0.58 ± 0.13 | 0.58 ± 0.09 |
| **3** | **dMHL** | 0.8 ± 0.08 | 0.71 ± 0.12 | 0.73 ± 0.11 | 0.72 ± 0.08 |
|  | **MPCI** | 0.82 ± 0.06 | 0.74 ± 0.11 | 0.74 ± 0.11 | 0.74 ± 0.06 |
|  | **MHL** | 0.74 ± 0.09 | 0.66 ± 0.12 | 0.69 ± 0.11 | 0.67 ± 0.08 |
| **4** | **dMHL** | 0.91 ± 0.06 | 0.82 ± 0.11 | 0.84 ± 0.1 | 0.83 ± 0.08 |
|  | **MPCI** | 0.92 ± 0.04 | 0.83 ± 0.08 | 0.85 ± 0.08 | 0.84 ± 0.05 |
|  | **MHL** | 0.86 ± 0.06 | 0.77 ± 0.1 | 0.78 ± 0.1 | 0.78 ± 0.07 |
| **5** | **dMHL** | 0.92 ± 0.04 | 0.82 ± 0.08 | 0.87 ± 0.08 | 0.84 ± 0.06 |
|  | **MPCI** | 0.96 ± 0.03 | 0.9 ± 0.07 | 0.91 ± 0.06 | 0.91 ± 0.04 |
|  | **MHL** | 0.9 ± 0.05 | 0.81 ± 0.09 | 0.82 ± 0.09 | 0.82 ± 0.06 |
| **10** | **dMHL** | 1 ± 0.01 | 0.95 ± 0.05 | 0.98 ± 0.04 | 0.96 ± 0.03 |
|  | **MPCI** | 1 ± 0 | 0.98 ± 0.03 | 0.99 ± 0.02 | 0.99 ± 0.02 |
|  | **MHL** | 0.99 ± 0.01 | 0.94 ± 0.06 | 0.97 ± 0.04 | 0.95 ± 0.04 |

(3) The authors consistently use 400 randomly selected regions for benchmarking. However, it is unclear how sensitive the results are to region selection. Given that MHL has been shown to perform optimally in MHB regions, could you repeat the analysis in Figure 2 using all MHB regions and compare the performance of MHL versus MPCI? Additionally, statistical significance tests are missing in Figure 2C and should be added.

We appreciate the reviewer’s important question regarding the sensitivity of our results to region selection and the potential advantage of MHL within Methylation Haplotype Blocks (MHBs).  First, we have added Wilcoxon rank-sum tests to the SVM classification results in Figure 2C, as suggested.

In response to performance on MHB blocks, we have conducted the same CD4 vs. CD8 classification analysis using MHB regions as defined by Guo et al. (Nature Genetics, 2017) and compared the results with those obtained from randomly selected regions. Table 6 demonstrates the results obtained on MHB regions, and Table 7 are the results obtained on analysis on random regions.

**Table 6: Analysis on MHB regions (*chr22 only, for computational efficiency*):**

| **Metric** | **MPCI (Mean)** | **MHL (Mean)** | **Difference** | **p-value** |
| --- | --- | --- | --- | --- |
| **AUC** | 0.975 | 0.905 | +0.070 | 0.027 (*) |
| **Accuracy** | 0.905 | 0.860 | +0.045 | 0.158 (NS) |
| **Sensitivity** | 1.000 | 0.930 | +0.070 | 0.006 (**) |
| **Specificity** | 0.810 | 0.790 | +0.020 | 0.688 (NS) |

**Table 7: Analysis on randomly selected regions (original analysis):**

| **Metric** | **MPCI (Mean)** | **MHL (Mean)** | **Difference** | **p-value** |
| --- | --- | --- | --- | --- |
| **AUC** | 0.915 | 0.700 | +0.215 | 2.04×10⁻⁷ (***) |
| **Accuracy** | 0.740 | 0.470 | +0.270 | 1.70×10⁻⁷ (***) |
| **Sensitivity** | 0.720 | 0.470 | +0.250 | 0.0013 (**) |
| **Specificity** | 0.760 | 0.470 | +0.290 | 1.46×10⁻⁵ (***) |

Key observations:

1. Both MPCI and MHL show improved performance in MHBs compared with random regions, which is expected given that MHBs are pre-defined regions with high methylation linkage disequilibrium (r² ≥ 0.5 between adjacent CpGs). This prior knowledge—selecting regions with strong CpG co-methylation—naturally enhances any metric designed to capture methylation consistency, including both MHL and MPCI.

2. MPCI still outperforms MHL even within MHBs, particularly in AUC (p = 0.027) and Sensitivity (p = 0.006), demonstrating its robustness even in regions optimized for MHL’s design.

3. The performance gap between MPCI and MHL is smaller in MHBs, indicating that MHL benefits disproportionately from the pre-filtered, high-co-methylation regions, as it is specifically designed to quantify fully methylated haplotype blocks.

We acknowledge that MHBs selectively include consecutive, highly correlated CpG sites—precisely the scenario where MHL is designed to excel. In contrast, randomly selected regions better represent the diverse methylation landscape encountered in real-world biomarker discovery, where regions may exhibit partial, heterogeneous, or inconsistent methylation patterns. Therefore, while both analyses are informative, we believe the random region comparison offers a more balanced and generalizable assessment of metric performance, particularly for applications where prior knowledge of optimal MHB regions is unavailable.

The improved performance of both metrics in MHBs highlights that region pre-selection based on co-methylation inherently boosts any consistency-based metric. This does not diminish MPCI’s advantage, but rather underscores that in unbiased, genome-wide contexts (simulated by random region selection), MPCI’s symmetric design provides substantially greater discriminative power.

We have added a short discussion on this to the revised version of the manuscript (results section - Performance of MPCI in biomarker discovery contexts), and included the above tables in the supplementary material (Supplementary Table 2).

(4) In Figure 3, since the samples are simulated, the authors should generate multiple independent cohorts (each with 100 healthy and 100 disease samples) rather than repeatedly resampling from a single cohort. This would provide a more robust assessment of performance variability.

We thank the reviewer for the valuable suggestion regarding the robustness of performance evaluation in our simulated experiments. In response, we have revised our analysis to incorporate both:

1. Generation of multiple independent simulated cohorts (as suggested here), and

2. Use of nested cross-validation (CV) to avoid overfitting and provide a reliable performance estimate.

We also have changed the simulation spike ratio to have more points below 5% to capture the differences in the small spike ratio percentages (metrics have similar near perfect performance above 5%). The new spike ratios are (1%, 2%, 3%, 4%, 5%, 10%),

Revised simulation design:

For each spike-in ratio we now generate 5 independent cohorts, each containing 100 healthy and 100 disease samples simulated from distinct random draws of the underlying read pools. This ensures that performance variability is assessed across truly independent datasets rather than repeated resampling from a single cohort.

Revised evaluation method:

For each independent cohort, we evaluate classifier performance using a nested CV scheme:

- Outer loop: 5-fold CV for train/test splits.

- Inner loop: 3-fold CV for hyperparameter tuning (SVM cost parameter).

- Performance metrics (AUC, accuracy, sensitivity, specificity) are aggregated across outer test folds and then averaged across the 5 independent cohorts.

Updated results:

Results show that using an integrated metric for methylation and unmethylation signals such as MPCI and dMHL has significant advantage over using MHL alone. MPCI demonstrated better performance at all spike ratios in comparison with MHL and dMHL. As the spike-in ratio increased, MPCI continued to outperform other metrics, achieving near-perfect performance at ratio of 10% . We have updated Figure 3, the Results section and Methods to reflect this improved simulation and evaluation framework. We believe these revisions strengthen the statistical validity of our study and thank the reviewer for their insightful feedback.

(5) In Figure 5B, it appears that dMHL could achieve a similarly high ranking as MPCI. This observation warrants further discussion.

The reviewer is correct in that for the specific region highlighted in Figure 5B, the differential behavior of MHL and uMHL suggests that the derived metric dMHL (MHL - uMHL) could achieve a ranking similar to MPCI. This point is well-taken and underscores the principle—central to our study—that effective region prioritization must consider both ends of the haplotype methylation spectrum.

Indeed, in this region, the oligodendrocyte sample shows a strong shift towards fully methylated haplotypes (high MHL), while the cfDNA samples maintains a distinct population of fully unmethylated haplotypes (high uMHL). Consequently, the arithmetic difference— dMHL — for oligodendrocyte samples is a large positive value and for cfDNA samples is a large negative value, which would grant it a similarly high rank if prioritized by dMHL alone. This example validates the importance of capturing shifts in both hyper- and hypomethylated haplotypes.

However, to assess whether dMHL is generally equivalent to MPCI for marker selection, we performed a systematic comparison. When we ranked all regions by dMHL and compared the resulting top 10 markers to the MPCI top 10, we found an intersection of only 6 regions (figure 6). This indicates that while the two metrics correlate, they are not practically identical for prioritization. We have revised our discussion in results section (Investigating DMR prioritization for disease detection) to clarify this distinction and to emphasize that considering both methylated and unmethylated haplotypes is crucial.

**Figure 6: Venn Diagram of top 10 markers detected using MPCI and dMHL**

MPCI top 10

dMHL top 10

4

4

6

We observed, for example, a region ranked 6th by MPCI that falls to 40th by dMHL (figure 7), and another ranked 4th by dMHL that plummets to 50th by MPCI ranking (figure 8). These large swaps in priority directly stem from how each metric interprets the underlying methylation haplotype patterns.

**Figure7: Region1 (MPCI rank = 6, dMHL rank = 40)**

**Figure 8: Region2 (MPCI rank = 50, dMHL rank = 4)**

(6) To properly benchmark cfDNA-based cancer detection, the authors should validate their approach using real cfDNA methylation datasets rather than relying solely on simulated data. Several public datasets are available for this purpose, and their inclusion would significantly strengthen the manuscript's conclusions.

We thank the reviewer for raising this important point regarding the need for validation on real cfDNA data. The main limitation we initially faced was regarding timely access to raw data from external cfDNA datasets. Fortunately we were able to identify and access a relevant and useful dataset for this validation during the revision. We have now conducted a new analysis using a whole-genome bisulfite sequencing (WGBS) dataset from a clinically relevant liquid biopsy context: pre- and post-liver transplant plasma cfDNA samples (GEO: GSE262275). While this dataset is not a cancer dataset, it presents a biologically analogous and stringent real-world scenario: detecting a minor fraction of tissue-specific DNA (donor liver DNA) within a background of recipient cfDNA. This mirrors the fundamental challenge in cancer liquid biopsy—identifying low-abundance, disease-derived methylation signatures amid a majority of healthy signal.

We selected 58 samples from the dataset (27 pre-transplant, 31 post-transplant day 0). Next, 400 genomic regions with high sequencing coverage on chromosome 22 were selected and MPCI and dMHL were calculated for each region. After quality filtering, classification was performed using a linear SVM with nested 5x3-fold cross-validation as described before. Performance was assessed via AUC, accuracy, sensitivity, and specificity, with statistical comparison using paired Wilcoxon tests (Benjamini-Hochberg corrected).

MPCI significantly outperformed dMHL, achieving:

- AUC: 0.961 ± 0.013 (vs. 0.934 ± 0.015 for dMHL)
- Specificity: 0.990 ± 0.010 (vs. 0.893 ± 0.042, p = 0.031)
- Accuracy: 0.868 ± 0.023 (vs. 0.768 ± 0.027, p = 0.014)
- Sensitivity: 0.791 ± 0.038 (vs. 0.692 ± 0.050)

This analysis has been added as a new subsection, “**Performance of MPCI in classifying real liquid biopsy experiments,”** in the revised manuscript and the results of classification is added to figure 3 (panel c and d).

We believe this new analysis significantly strengthens our conclusions regarding MPCI’s practical utility in liquid biopsy applications, and we sincerely thank the reviewer for their constructive suggestion.
