# Supplementary material for: MPCI: A novel metric for quantifying DNA methylation patterns in NGS data
Source: PLoS Comput Biol. 2026 Mar 24;22(3):e1014076. doi: 10.1371/journal.pcbi.1014076 (PMC13035127; doi:10.1371/journal.pcbi.1014076)
Supplement: Attachment — Submitted filename: 02-23-2026_Response_to_Reviewers.docx [file pcbi.1014076.s010.docx]

Response to Reviewers

We would like to thank the Reviewers for their time and effort in evaluating our revised manuscript. We are pleased that both Reviewers found that we had adequately addressed their previous concerns. We appreciate the remaining insightful comments from Reviewer 2, which we have addressed below. These suggestions have helped us improve the clarity and reproducibility of our work.

Reviewer 1: The authors addressed the previously raised issues.

Thank you for your positive feedback and for reviewing our revised manuscript.

Reviewer 2: My comments were largely addressed. However, I found that the authors did not describe the tools used in the analysis or the parameters employed. For example, it is unclear how different read-level metrics were calculated; the authors should describe the tools for alignment, read filtering, and coverage thresholds in detail in the Methods section. In addition, the authors should make all their scripts publicly available to ensure reproducible research. Currently, I only see the R function for MPCI calculation.

We thank the reviewer for acknowledging that our previous revisions addressed their major concerns. We also thank them for highlighting the need for greater clarity regarding our bioinformatics pipeline and for emphasizing the importance of reproducibility. We agree with these points entirely. To address this, we have made the following modifications to the manuscript and our supporting materials:

1. Detailed Description of Data Source, File Format, and Metric Calculation in the Methods Section:

We have expanded the "Data collection, processing and metric calculation" subsection of the Methods to provide more detailed account of our analysis pipeline. The revised section now clarifies that we downloaded PAT files from public repositories, describes the file format used in our analysis, and details how the methylation heterogeneity metrics were calculated. The PAT file format stores read-level DNA methylation information and serves as a compact methylation-oriented representation of a BAM file. Because these files contain read-level methylation patterns rather than raw sequencing reads, no additional alignment, read filtering, or processing of FASTQ files was required for our analysis. The section now looks as follows (the added parts are highlighted):

Data collection, processing and metric calculation

We obtained a comprehensive dataset of Whole Genome Bisulfite Sequencing (WGBS) from GSE186458 comprising methylation profiles from various tissues (27). In the original study, paired-end FASTQ files were mapped to the human (hg19 and hg38), lambda, pUC19 and viral genomes using bwa-meth (v.0.2.0) (31) then converted to BAM files using SAMtools (v.1.9) (32). Duplicated reads were marked by Sambamba (v.0.6.5) (33), and reads with low mapping quality, duplicates, or those not mapped in a proper pair were excluded. Reads were then stripped of non-CpG nucleotides and converted to PAT format using wgbstools (v.0.1.0) (34). For our analysis, we directly downloaded these processed PAT files from GEO (27). Multiple differential methylation analysis scenarios were considered using this dataset. For the evaluation of cell type classification contexts, blood T cell (CD4 and CD8), brain (oligodendrocyte, cortex, neuron and cerebellum), liver, lung, colon and bone marrow samples were selected from this dataset.

To simulate liquid biopsy scenarios, in-silico spike-in experiments were conducted using WGBS data from healthy cfDNA and neuron samples obtained from the same dataset (27). Reads from target tissue were randomly selected, and a percentage of reads (1%, 2%, 3%, 4%, 5%, and 10%) were substituted with reads from the healthy cfDNA. This approach mimicked varying levels of disease-specific cfDNA in a background of healthy cfDNA, allowing for the evaluation of the proposed metric under conditions representative of real-world liquid biopsy applications.

Our evaluations used 400 randomly selected regions with diverse range of CpG numbers (100 regions: 3 to 7 CpGs, 100 regions: 8 to 12 CpGs, 100 regions: 13 to 17 CpGs, 100 regions: 18 to 22 CpGs). 8 regions with genomic overlap were filtered from the downstream analysis. These regions were selected to ensure a robust evaluation of the proposed metric.

To evaluate MPCI in real liquid biopsy data, cell-free DNA methylation data from liver transplant patients were obtained from GEO accession GSE262275 (29), comprising 130 blood samples from 44 patients at various timepoints. For this dataset, the original study's processing included trimming paired-end FASTQ files with TrimGalore (v.0.6.6) (35) and mapping them to the human genome (GRCh37/hg19) using Bismark (v.0.22.3) (36). The resulting BAM files were processed with SAMtools (v.1.12) (32), and reads were subsequently converted to PAT format using wgbstools (v.0.1.0) (34). We downloaded these pre-computed PAT files from GEO and focused on pre-transplant (POD0-PRE, n=27) and post-operative day 0 (POD0-POST, n=31) samples and performed our analysis on the 400 most covered regions in the dataset (see performance evaluation subsection).

All WGBS data were therefore downloaded from GEO in PAT file format (27, 29). The PAT file format stores read-level DNA methylation information and serves as a compact methylation-oriented representation of a BAM file. Each row in a PAT file contains the chromosome, starting CpG index, a methylation pattern string across consecutive CpG sites (with C for methylated, T for unmethylated, and '.' for unknown status), and the count of reads exhibiting that exact pattern.

Using these PAT files as input, we calculated the key methylation metrics used in our analysis. The methylation quantifying metrics including Methylation Haplotype Load (MHL), Methylation Entropy (ME), Epi-polymorphism (EP), Proportion of Discordant Reads (PDR), Fraction of Discordant Read Pairs (FDRP), and Quantitative FDRP (qFDRP) were computed following the mathematical formulations described by (19). Unmethylation Haplotype Load (uMHL) was calculated based on same mathematical formulation of MHL adjusted for fully unmethylated haplotypes. MPCI, our introduced metric, was calculated using a custom R function that implements the formula (see MPCI metric definition subsection). R script for the calculation of all the metrics is deposited in https://github.com/NaghmeNazer/MPCI/tree/main/cell_type_classification_contexts .

2. Improved Public Availability of All Scripts on GitHub:

We fully agree with the reviewer that making all code publicly available is essential for reproducible research. While we had previously made all custom scripts available in our GitHub repository, we acknowledge that our initial repository structure, with scripts distributed across branches, was not optimally user-friendly. We have now completely reorganized our GitHub repository. All custom scripts used in the study have been consolidated into the main branch, making the repository significantly more accessible and intuitive for users.

The repository is now publicly accessible at:

https://github.com/NaghmeNazer/MPCI

We believe these additions thoroughly address the reviewer's concerns and significantly strengthen the manuscript's transparency and reproducibility. We are grateful for the suggestions.
